# Chronic inflammatory demyelinating polyneuropathy with pulmonary nocardiosis: A case report

**DOI:** 10.1097/MD.0000000000038544

**Published:** 2024-06-14

**Authors:** Cheng Yan, Ting-Ting Liu, Li-Tao Gao

**Affiliations:** a Department of Clinical Pharmacy, Bethune International Peace Hospital, Shijiazhuang, Hebei, China; b Department of Neurology, Bethune International Peace Hospital, Shijiazhuang, Hebei, China.

**Keywords:** adverse drug reaction, chronic inflammatory demyelinating polyneuropathy, immunosuppressant, pulmonary nocardiosis

## Abstract

**Rationale:**

Chronic inflammatory demyelinating polyneuropathy (CIDP) is an immune-mediated motor sensory peripheral neuropathy that is rare in clinical practice. This treatment method aims to suppress potential immunopathology. Nocardiosis is a rare, destructive, opportunistic disease. We report a case of failed treatment of CIDP combined with pulmonary nocardiosis, and for the first time, we link these 2 diseases together.

**Patient concerns:**

A 65-year-old man developed symmetrical limb weakness. Four months later, he was diagnosed with CIDP and started receiving glucocorticoid (GC) treatment. The disease progressed slowly and was treated with mycophenolate mofetil (MMF) in combination. He did not follow the doctor requirements for monthly follow-up visits, and the preventive medication for sulfamethoxazole/trimethoprim was not strictly implemented. Two months after the combination therapy, the patient developed fever, coughing and sputum production, as well as fatigue and poor appetite. Based on imaging and etiological results, he was diagnosed with pulmonary nocardiosis.

**Diagnoses:**

Chronic inflammatory demyelinating polyneuropathy, pulmonary nocardiosis.

**Interventions:**

After treatment with antibiotics, the patient lung infection temporarily improved. However, the patient CIDP condition progressed, limb weakness worsened, respiratory muscle involvement occurred, and intravenous immunoglobulin (IVIG) was administered. However, there was no significant improvement in the condition, and the patient died.

**Outcomes:**

In this report, we present a case of a patient with CIDP and pulmonary nocardiosis. It is worth noting that in order to avoid the progression and recurrence of CIDP, we did not stop using related therapeutic drugs during the treatment process, the patient had repeatedly refused to use IVIG. Despite this, the patient condition worsened when lung inflammation improved, leading to persistent respiratory failure and ultimately death. Treatment contradictions, medication issues, and patient compliance issues reflected in this case are worth considering.

**Lessons:**

For patients with CIDP receiving immunosuppressive therapy, attention should be paid to the occurrence and severity of Nocardia infection. Therefore, early detection and treatment are necessary. We need to pay attention to the compliance of patients with prophylactic use of antibiotics, strengthen the follow-up, and urge them to return to their appointments on time.

## 1. Introduction

Chronic inflammatory demyelinating polyneuropathy (CIDP) is an immune-mediated disease of the peripheral nervous system. The main clinical manifestations are symmetrical weakness and sensory dysfunction in the proximal and distal limbs. The course of the disease often presents as chronic progression or remission recurrence, seriously affecting the physical and mental health of patients.^[1,2]^ At present, the factors triggering the onset and progression of CIDP are not clear, and treatment methods aim to suppress potential immunopathology.^[3]^ Glucocorticoids (GC), intravenous immunoglobulins (IVIG), and plasma exchange (PE) are the first-line therapies. Immunosuppressive or immunomodulatory drugs, such as azathioprine, mycophenolate mofetil (MMF), and rituximab, are adjunctive drugs chosen to reduce the dosage of first-line drugs. When first-line drugs are ineffective, they can also serve as substitutes.^[4]^ Nocardiosis is a rare and destructive opportunistic disease that tends to occur in patients with impaired immune function.^[5]^ Here, we report a case of CIDP combined with pulmonary nocardiosis and, for the first time, link these 2 diseases together. It is worth noting that in order to avoid the progression and recurrence of CIDP, we did not stop using immunomodulatory drugs during the treatment process. Despite this, the patient condition worsened when lung inflammation improved, leading to persistent respiratory failure and ultimately death. Treatment contradictions, medication issues, and patient compliance issues reflected in this case are worth considering.

## 2. Case report

**Figure 1.**
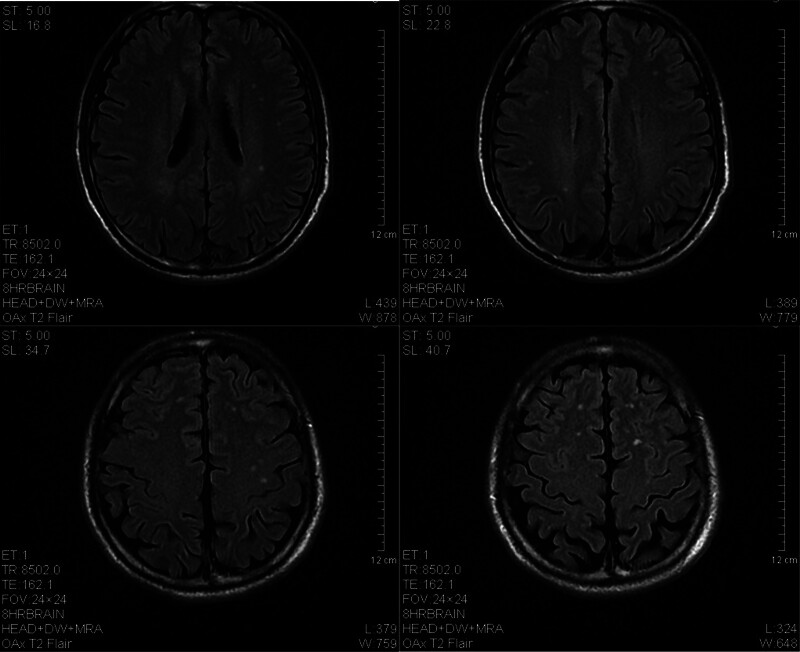
Brain magnetic resonance imaging of the patient (T2 Flair).

**Figure 2.**
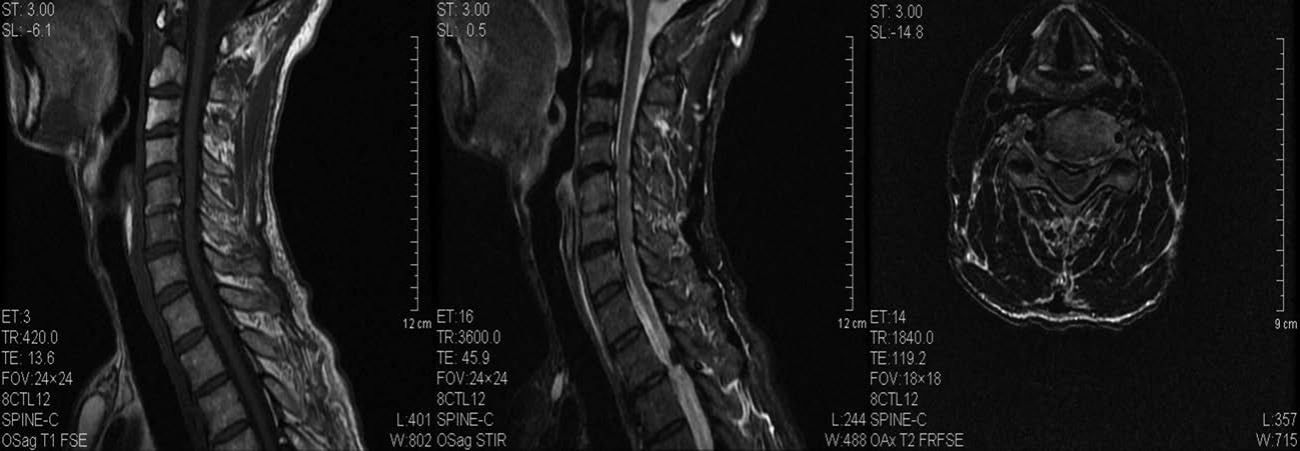
The patient cervical magnetic resonance imaging results.

**Figure 3.**
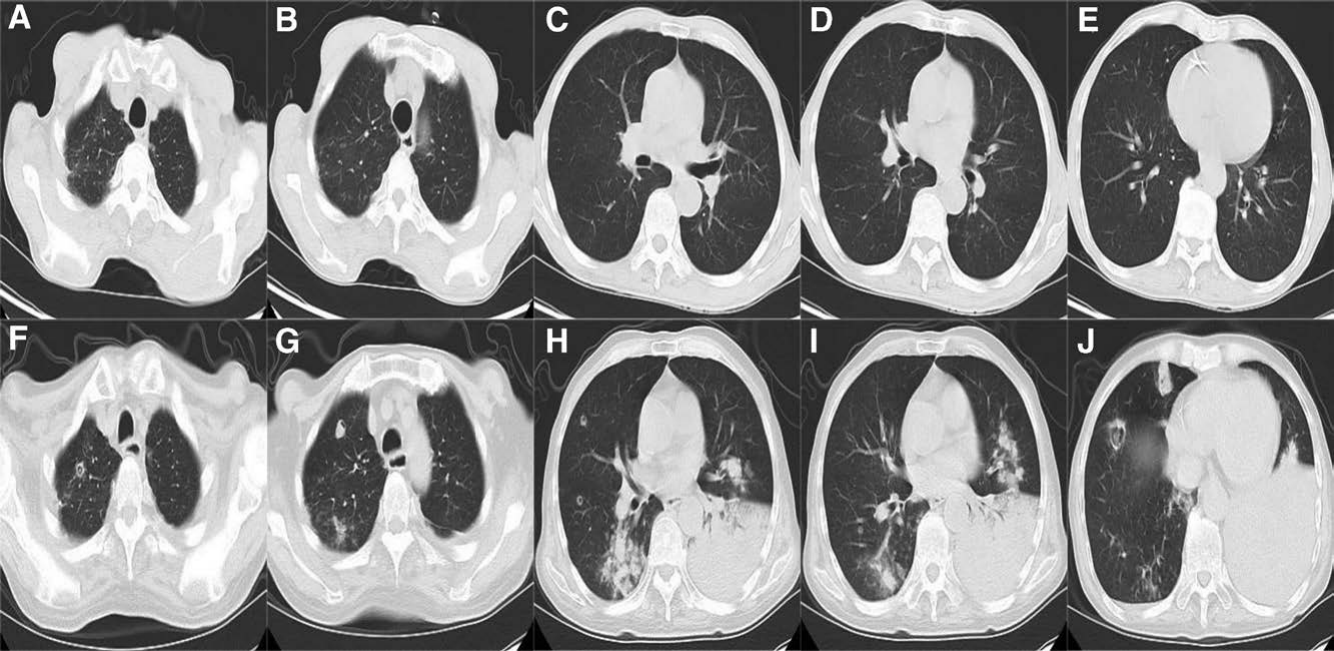
Patient lung computed tomography results. (A–E) Images were taken in June 2023. (F–J) Images were taken in November 2023.

**Figure 4.**
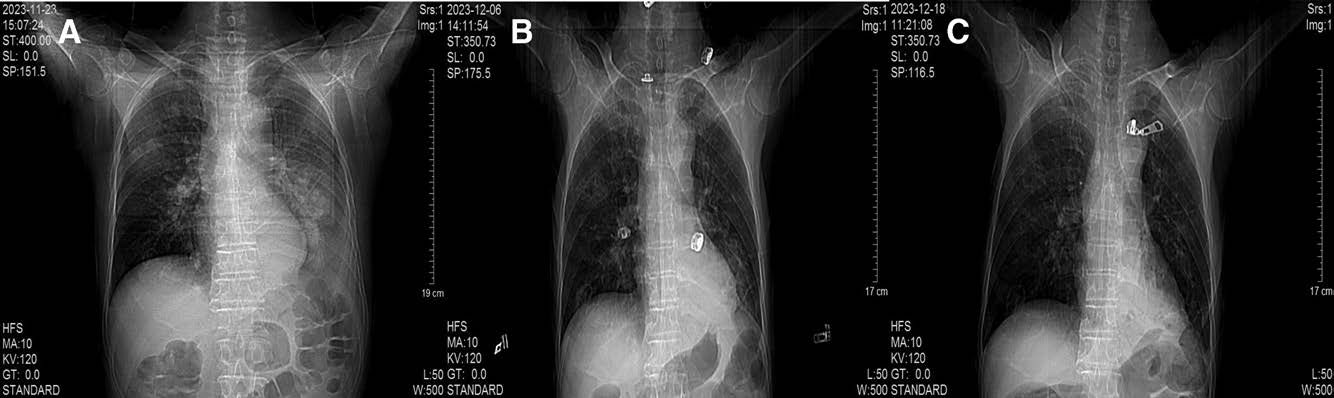
Comparison of 3 lung computed tomography results of the patient. (A) November 23rd. (B) December 6th. (C) December 18th.

The patient was a 65-year-old man who developed symmetrical limb weakness without an obvious cause in February 2023; he paid no attention. In June 2023, he further developed numbness at the distal ends of both upper limbs and came to our hospital for treatment. Electromyography revealed extensive neurogenic abnormalities involving the cervical, thoracic, lumbar, and medullary segments. The left extensor digitorum brevis branch of the common peroneal nerve showed complete damage, while the rest showed incomplete damage. A small number of tested sensory nerve conduction velocities were within the normal lower limit, while some tested motor nerve conduction velocities slowed down and compound muscle action potential amplitudes decreased. The myositis antibody spectrum was negative. Brain magnetic resonance imaging (MRI) revealed multiple chronic ischemic changes in the bilateral paraventricular white matter and frontal lobe, with mild brain atrophy (Fig. 1). Cervical MRI indicated spondylosis with disc herniation in the 3 to 4, 4 to 5, 5 to 6, 6 to 7 cervical vertebrae and partial fusion in the 5 to 6 cervical vertebrae. The spinal cord signal was uniform and there were no abnormal signs in the accessory and paravertebral soft tissues (Fig. 2). Cerebrospinal fluid examination showed protein at 0.77 g/L, Pandy test (+), and nucleated cells (0/µL). The limb muscle strength was level 4, with symmetrical weakening of the bilateral biceps, triceps, radial membrane, and knee Achilles tendon reflexes. He was diagnosed with CIDP and received methylprednisolone shock therapy and long-term oral administration (40 mg qd), while also taking methylcobalamin and vitamin B1 tablets, after which the patient symptoms improved. In September 2023, the patient experienced worsening limb weakness, accompanied by numbness and pain at the distal end of the limbs. Electromyography showed peripheral neuropathy, involvement of motor and sensory fibers, and axonal lesions combined with demyelinating lesions. We administered methylprednisolone shock therapy again, pregabalin capsules, and MMF capsules (500 mg, bid). The patient was discharged after his symptoms had improved. In November 2023, the patient condition worsened again, with significant weakness in both lower limbs, difficulty in standing and walking, and an inability to stand up after squatting. In addition, he developed a fever with a body temperature as high as 38.5°C, significant fatigue, and poor appetite. On November 22nd, the patient was admitted to our hospital for treatment. He coughed with a white sticky phlegm accompanied by shortness of breath. Auscultation showed weakened breathing sounds in the left lung and moist rales in both lungs. The muscle strength of both upper limbs was level 5, whereas that of the lower limbs was level 4, and the muscle tension was normal. Pathological signs were not observed. Computed tomography (CT) of the lung showed patchy consolidation shadows in the lower left lung, with multiple patchy and nodular high-density shadows in both lungs; cavities could be seen within some lesions (Fig. 3). Blood gas analysis indicated type I respiratory failure, and drugs such as meropenem and linezolid were administered for anti-infection treatment. On November 27th, sputum culture revealed Nocardia, and a metagenomic next-generation sequencing test of bronchoalveolar lavage fluid reported Nocardia gelsenkirchen. The patient was diagnosed with pulmonary nocardiosis, and sulfamethoxazole/trimethoprim (SMX/TMP) was administered to treat the infection. After treatment, the patient respiratory symptoms improved, significant absorption of bilateral lung lesions was observed underwent 3 lung CT scans after being hospitalized in November, November 23rd, December 6th, and December 18th (Fig. 4). Based on the imaging results, the patient had the most severe infection on November 23rd and gradually improved. Respiratory symptoms also improved, and he was able to get out of bed and move around. On December 20th, the patient suddenly developed a cough and weakness accompanied by dyspnea, and mechanical ventilation was required. The patient limb weakness worsened; the muscle strength of both upper limbs was level 1, and that of both lower limbs was 0. It was considered a progression of the primary disease, and muscle weakness led to aggravated respiratory failure. This was clearly not caused by the gradual worsening of the infection, as the imaging and clinical symptoms reflecting the severity of the infection were gradually improving. Moreover, from an imaging perspective, the patient did not require mechanical ventilation during the most severe infection on November 23, but had to go on a ventilator on December 20, which also confirmed that sudden respiratory muscle paralysis and worsening limb weakness were caused by CIDP. The patient underwent tracheostomy and assisted breathing, and IVIG (400 mg/kg/d for 5 days) was administered. However, there was no significant improvement in the condition, and the patient died. To avoid progression and recurrence of CIDP, the patient did not stop using MMF or methylprednisolone during hospitalization. Many other antibacterial drugs such as amikacin and moxifloxacin have also been used. The exact antimicrobials and their duration of administration are shown in Tab. 1.

**Table 1:** The use of antibiotics in the patient diagnosed with pulmonary nocardiosis.

Drug	Daily dose	Duration of administration
Cefuroxime	4.5g	11.22–11.23
Meropenem	3g	11.24–12.07;12.23–12.27
Linazolamide	1.2g	11.24–12.27
SMX/TMP	1600/320mg	11.27–12.27
Amikacin	0.8g	12.08–12.16
Moxifloxacin	0.4g	12.08–12.16
Ceftriaxone	2g	12.17–12.22
Polymyxin B	100wu	12.23–12.27

## 3. Discussion

CIDP is a type of motor sensory peripheral neuropathy mediated by immunity. Studies in the literature have estimated various prevalence rates, ranging from 1 to 9 per 1000,000 people.^[6,7]^ Diagnosis relies mainly on clinical symptoms, signs, and electrophysiological examinations. Owing to its rarity in clinical practice and the variability of its symptoms, it is highly susceptible to misdiagnosis and mistreatment in clinical practice. Patients with CIDP typically experience symptoms that progress for more than 8 weeks, with chronic progression or relief of recurrence, including varying degrees of limb weakness, mostly symmetrical, involving both proximal and distal ends, with reduced or absent limb key reflexes, accompanied by deep and superficial sensory abnormalities; albuminocytologic dissociation can be observed in cerebrospinal fluid examination; electrophysiological examination indicates a slowdown in peripheral nerve conduction velocity, conduction block, or abnormal waveform dispersion.^[1,2,6]^ The diagnostic process of this patient is complex, and currently, CIDP remains an exclusive diagnosis. Before diagnosing CIDP, other diseases should be excluded. In June 2023, we conducted a series of examinations and tests on the patient and inquired in detail about his onset process and family history. The patient denied having a history of genetic, infectious, or special diseases in his family, and there were no similar patients in his family. No obvious abnormalities were observed in routine blood tests, blood coagulation, erythrocyte sedimentation rate, hepatitis B, hepatitis C, syphilis, acquired immunodeficiency syndrome, rheumatoid factor, immunoglobulin M, immunoglobulin E, immunoglobulin A, immunoglobulin G, antinuclear antibody, blood complement C3, blood complement C4, and autoimmune antibodies. The patient had been taking atorvastatin for a long time to regulate blood lipids. Muscle damage related to atorvastatin is mainly characterized by myalgia and myasthenia, which are usually transient. The patient was mainly characterized by myasthenia, did not have myalgia, and the course of the disease was chronic progression; therefore, drug poisoning was ruled out. Ischemic cerebrovascular disease often presents as hemiplegia and hemiblindness, accompanied by dizziness or consciousness disorders, and imaging examination shows ischemic lesions. The patient had symmetrical limb weakness, which could be ruled out using cranial MRI. Multiple sclerosis presents as alternating episodes of limb weakness accompanied by sensory abnormalities and is usually accompanied by visual changes. Characteristic multiple lesions can be seen on cranial MRI. Our patient did not show any visual changes, and multiple sclerosis was ruled out based on the MRI results. Paraneoplastic peripheral neuropathy is mostly purely sensory or sensorimotor with obvious sensory symptoms and progressive progression. Peripheral nerve damage can occur before, simultaneously, or after the onset of cancer. The patient cranial MRI, cervical MRI, pelvic MRI, and chest CT did not show any evidence of tumor-related symptoms. The clinical manifestations of our patient were mainly motor symptoms, albuminocytologic dissociation was observed in the cerebrospinal fluid examination, and paraneoplastic peripheral neuropathy was ruled out. Polymyositis is characterized by progressive weakness of the symmetrical proximal muscles of the limbs, and electromyography indicates active myogenic damage. The patient muscle weakness symptoms were simultaneously affected at the proximal and distal ends, and electromyography showed extensive neurogenic abnormalities. The myositis antibody spectrum was negative and polymyositis was ruled out. After excluding these diseases, the patient was diagnosed with CIDP. The pathological process of primary demyelination leads to secondary axonal degeneration, which results in disability. Early diagnosis and treatment can minimize the severity of axonal degeneration. Our patient came to the hospital for treatment nearly 4 months after experiencing symptoms, which delayed the start of treatment. CIDP can be divided into 2 categories: typical and variant. A typical CIDP possesses all the above symptom characteristics, while variant CIDP includes pure motor type, pure sensory type,distal acquired demyelinating symmetric polyneuropathy, and multifocal acquired demyelinating sensory and motor neuropathy.^[8,9]^ The patient developed symptoms at 65 years of age and presented with chronic progression, meeting the typical diagnostic criteria for CIDP. GC, IVIg, or PE can be used as first-line treatment for CIDP. We considered several factors when choosing treatment options, such as safety, accessibility, and disease characteristics. At present, with faster onset and better compliance than GC,IVIg tends to be the preferred treatment for most patients, but the cost is high. PE is only used for IVIG- and GC-refractory patients because of the lack of long-term data and equipment requirements. Owing to economic reasons, our patient chose GC as the initial treatment. We observed that methylprednisolone therapy was effective and the patient symptoms improved; however, over time, the CIDP condition had progressed. Therefore, we added MMF, and the patient condition improved. Unfortunately, 2 months later, the patient developed nocardiosis.

Nocardiosis is an uncommon infectious disease caused by Gram-positive bacteria. Most infected people have low immune function accompanied by cell-mediated abnormalities, such as malignant tumors, organ and hematopoietic stem cell transplantation, and human immunodeficiency virus infection. Nocardia includes more than 90 species of bacteria, of which at least 54 are pathogenic to humans. Nocardia farcinica is the most frequently detected Nocardia species in China (24.5%), followed by Nocardia gelsenkirchen (20.8%).^[10–12]^ The most common way for Nocardia to enter the body is through inhalation, which can often invade the lungs and the central nervous system. Pathogen clearance in pulmonary Nocardia is difficult, and the treatment course is long. SMX/TMP is the main drug of choice, which can be combined with linezolid, ceftriaxone, amikacin, imipenem, and doxycycline.^[11,13]^ It is usually recommended to use 2 to 3 drugs intravenously for the initial treatment for 3 to 6 weeks. After significant improvement in the condition, oral medication should be considered for further 6 to 12 months.^[14,15]^ Due to the lack of specificity in the clinical symptoms of pulmonary nocardiosis, it is necessary to strengthen vigilance against high-risk populations, such as those using immunosuppressants or with chronic structural lung disease. Our patient was treated with a combination of GC and MMF for 2 months, after which patient developed fever, coughing, and sputum production. After a series of examinations, the final diagnosis was pulmonary nocardiosis. In terms of imaging, the most common forms of pulmonary nocardiosis are newly developed cavities and solid lesions. Pathogenic results are the gold standard for diagnosis, and the overall sensitivity of cultured respiratory or tissue samples is approximately 85% to 95%.^[16]^ With the rapid development of molecular detection technology, Nocardia can be quickly detected in relevant samples and even identified to the species level, with higher sensitivity than conventional culture. Therefore, when considering the possibility of nocardial infection, pathogenesis-related testing should be carried out on the primary site as soon as possible. After confirmation of pulmonary nocardiosis, treatment was positive. The combined application of multiple antibacterial drugs, such as SMX/TMP, meropenem, and linezolid, combined with sputum aspiration and alveolar lavage, improved the patient respiratory symptoms, and significant absorption of bilateral lung lesions was observed. Looking back at the treatment process, when the patient was diagnosed with pulmonary nocardiosis, we considered stopping the use of GC and MMF to control the infection as soon as possible. However, we were concerned that stopping the medication would lead to the progression and recurrence of CIDP, which is a contradiction. The patient presented with CIDP-related symptoms in February 2023 and progressed multiple times in June, September, and November, indicating that the current drug treatment was not effective. IVIG is also the first-line treatment option for CIDP. We repeatedly recommended that our patient use IVIG; however, because of its high cost, he refused. When the patient developed pneumonia, pulmonary nocardiosis was very dangerous, and immunosuppressants could make the infection more difficult to control. We believe that stopping the use of immunosuppressants and switching to IVIG treatment is beneficial and necessary. Therefore, we once again suggested that the patient discontinue immunosuppressive agents and switch to IVIG, but he still refused. Given the multiple progressions of the patient CIDP condition in recent months, we believe that continuing to use therapeutic drugs was also very necessary, and even considered increasing the dosage, which was a difficult decision. Despite the use of immunosuppressants, there was still significant improvement in infection and respiratory symptoms under the action of multiple antibiotics, indicating the effectiveness of anti-infection treatment. The patient CIDP suddenly progressed again on December 20th, endangering his life. He finally agreed to use IVIG, but unfortunately, everything was too late.

The patient was diagnosed with CIDP in June 2023, and a long-term oral methylprednisolone treatment plan (40 mg qd) was determined. High-doses of corticosteroids have profound effects on the distribution and function of neutrophils, monocytes, and lymphocytes. The NCCN Clinical Practice Guidelines in Oncology recommend using SMX/TMP for the prevention of Pneumocystis carinii pneumonia if the prednisone equivalent of steroids is 20 mg or more per day for 4 weeks or longer.^[17]^ Studies have also indicated that the preventive use of SMX/TMP is significantly associated with a reduced risk of nocardiosis.^[18,19]^ Therefore, we required the patient to visit our hospital for a follow-up visit 4 weeks after discharge to evaluate the patient opportunistic infection situation and add the use of SMX/TMP. Unfortunately, the patient did not return immediately. In September 2023, the patient CIDP condition worsened and he returned. MMF capsules were then added. Due to the lack of SMX/TMP in our hospital, we required the patient to purchase this drug outside of the hospital and received prophylactic medication at a dose of 800/160 mg thrice weekly. We informed our patient to undergo monthly follow-up and paid attention to symptoms such as fever, dry cough, and shortness of breath. If any symptoms of discomfort occur, medical attention is immediately sought. The patient was still not followed up on time. In early November, the patient CIDP condition worsened again, and he did not visit our hospital until he developed high fever. The patients’ multiple visits reflected poor patient compliance. Whether he actually took preventive medication according to our requirements and the response we received was also vague.

The patient treatment experience was valuable, and the lessons learned were heartbreaking. Research on the use of antibiotics for the prevention of nocardiosis has mostly been conducted in solid organ transplant recipients, who often use immunosuppressants for extended periods.^[18–20]^ Peleg et al identified 3 risk factors for nocardiosis: high-dose steroid use, high levels of calcineurin inhibitors 1 month prior to diagnosis, and cytomegalovirus disease 6 months prior to diagnosis.^[21]^ Coussement et al found that low-dose SMX/TMP could prevent pulmonary sporidiosis but not nocardiosis.^[20]^ Yetmar et al believed that the effectiveness of preventive medication may be related to the dosage or frequency of SMX/TMP.^[19]^ Passerini et al found that SMX/TMP prophylactic medication may reduce the risk of developing nocardiosis in solid organ transplant recipients. However, the optimal dose of SMX/TMP, which is considered to exert a protective effect, has not been determined.^[18]^ In contrast, in this meta-analysis, 66 out of 260 patients with nocardiosis (25.4%) were taking SMX/TMP. This breakthrough infection seems to be unrelated to the resistance of Nocardia to SMX/TMP, as most breakthrough infections are caused by isolates sensitive to SMX/TMP, which alleviates potential concerns about the progression of bacterial resistance caused by prophylactic drugs. It is worth noting that the fact that patients have prophylactic medication prescriptions for SMX/TMP does not necessarily mean that they are actually receiving SMX/TMP, and noncompliance may be another issue. Our view is that the use of SMX/TMP as a treatment option, even in breakthrough infections, is a valid option, and current prophylaxis should not discourage its use. In China, the recommended therapeutic dose for SMX/TMP is 1600/320 mg daily, and the preventive dose is 800/160 mg thrice weekly. Notably, SMX/TMP can cause adverse reactions such as granulocytopenia, thrombocytopenia, aplastic anemia, liver damage, and kidney damage. Routine blood, liver, and kidney function should be regularly monitored during medication.

CIDP is caused by macrophage-mediated inflammatory demyelination involving the proximal greater than the distal nerve segments, and the exact immune mechanisms are unknown.^[22]^ GCs have been shown to have different effects on T lymphocytes, and high-doses can lead to rapid depletion of circulating T-cells by redistributing them to other body regions. In addition, they inhibit interleukin-2, a cytokine essential for the function, differentiation, and proliferation of T-cells. They can also induce apoptosis of T lymphocytes, which further depletes the total pool of mature functioning T-cells.^[23]^ MMF inhibits inosine monophosphate, a rate-limiting enzyme in the de novo synthesis of guanosine nucleotides. Lymphocytes are more dependent on this pathway than other cells. Therefore, the cytotoxic effect of MMF is mediated by the inhibition of T- and B-lymphocyte proliferation and suppression of humoral immune responses by B-lymphocytes.^[24–26]^ All of these mechanisms result in a profound immunosuppressive state that promotes the development of opportunistic infections. The mechanism of action of IVIG is different from the above 2 drugs. Autoimmune diseases of the peripheral nervous system have so far been treated with exogenous high-dose IVIG, that act through several mechanisms including neutralization of pathogenic autoantibodies, modulation of lymphocyte activity, interference with antigen presentation, and interaction with fragment crystallizable receptors, cytokines, and the complement system.^[27]^ Interestingly, multiple studies have confirmed that the application of IVIG could reduce the incidence and mortality of infections in individuals with immune abnormalities.^[28–31]^ Therefore, we speculate that our patient might have had different treatment outcomes if immunosuppressive drugs were switched to IVIG as early as possible. IVIG is also a first-line treatment option, and in theory, the use of IVIG is not only beneficial for the control of CIDP, but also reduces the mortality rate of infected patients.

## 4. Conclusions

CIDP and pulmonary nocardiosis are clinically rare. We describe a case of failed treatment for CIDP combined with pulmonary nocardiosis, which is the first time that these 2 diseases have been linked together. Our lesson is that for patients with CIDP receiving immunosuppressive therapy, attention should be paid to the occurrence and severity of Nocardia infection. Therefore, early detection and treatment are necessary. We need to pay attention to the compliance of patients with prophylactic use of antibiotics, strengthen the follow-up, and urge them to return to their appointments on time.

## Author contributions

**Conceptualization:** Cheng Yan.

**Data curation:** Cheng Yan.

**Formal analysis:** Cheng Yan.

**Investigation:** Cheng Yan.

**Methodology:** Cheng Yan.

**Project administration:** Cheng Yan.

**Resources:** Li-Tao Gao.

**Software:** Ting-Ting Liu.

**Supervision:** Cheng Yan.

**Validation:** Cheng Yan.

**Visualization:** Ting-Ting Liu.

**Writing – original draft:** Cheng Yan.

**Writing – review & editing:** Li-Tao Gao.
